# Supplementary material for: CD146+ Endometrial-Derived Mesenchymal Stem/Stromal Cell Subpopulation Possesses Exosomal Secretomes with Strong Immunomodulatory miRNA Attributes
Source: Cells. 2022 Dec 10;11(24):4002. doi: 10.3390/cells11244002 (PMC9777070; doi:10.3390/cells11244002)
Supplement: Supplementary file 1 [file cells-11-04002-s001.zip › cells-2050850-supplementary.pdf]

cells MDPI

Supplementary Material

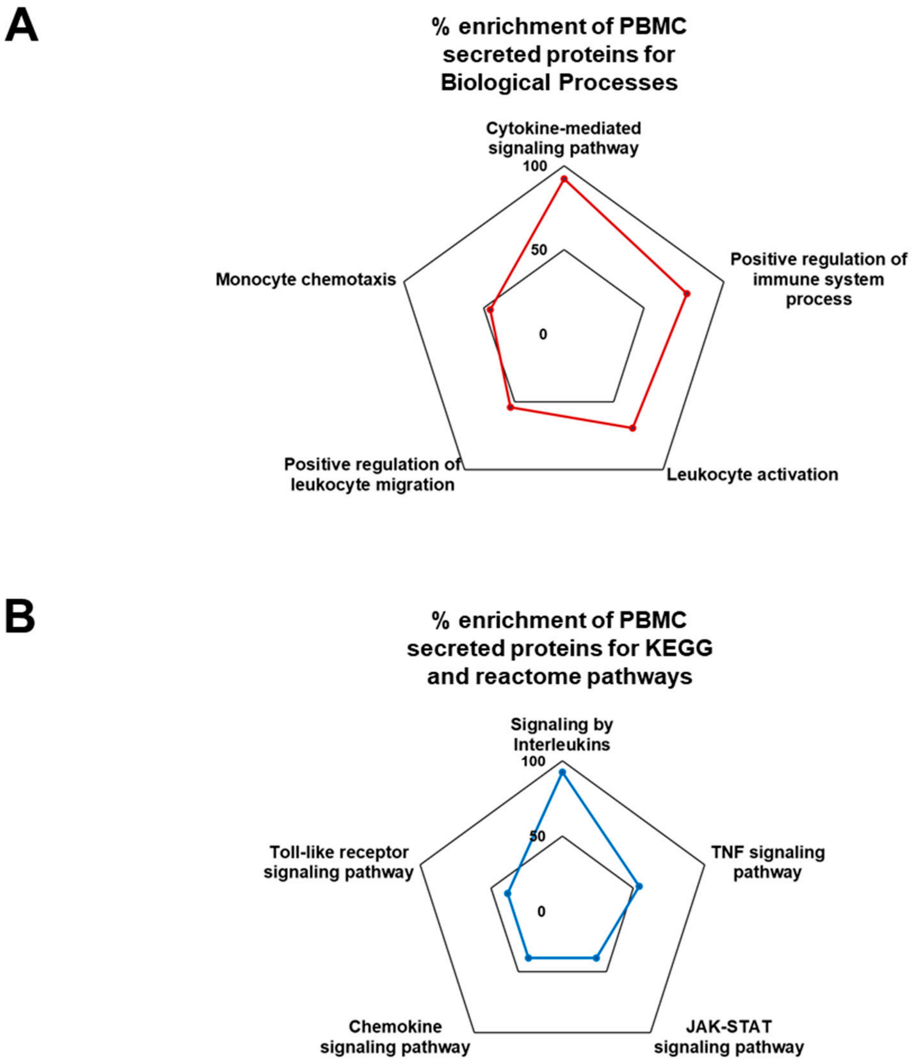

Supplementary Figure S1. Percentage (%) enrichment of PBMC secreted proteins for biological processes (A), KEGG, and reactome pathways (B).
